# Gray matter volume alterations in adolescents with ADHD are associated with cell type-specific transcriptional signatures

**DOI:** 10.3389/fneur.2026.1815799

**Published:** 2026-05-29

**Authors:** Meng Chen, Renhao Zhang, Zhongtian Chen, Kai Wu, Hui Wang, Haitao Yu

**Affiliations:** 1 Department of Medical lmaging, Luoyang Maternal and Child Health Hospital, Luoyang, China; 2 Department of Neurosurgery, First Affiliated Hospital of Bengbu Medical College, Bengbu, Anhui, China; 3 Department of Pediatric Surgery, Luoyang Maternal and Child Health Hospital, Luoyang, China; 4 Department of Pediatrics, Huangshan City People's Hospital, Huangshan City, China

**Keywords:** attention-deficit/hyperactivity disorder, enrichment analyses, gray matter volume, machine learning, transcriptional

## Abstract

**Objective:**

Attention-deficit/hyperactivity disorder (ADHD) is characterized by atypical brain development, yet the molecular and cellular mechanisms underlying its characteristic gray matter volume (GMV) alterations remain poorly understood. This study aimed to integrate neuroimaging and transcriptomic data to identify cell-type-specific transcriptional signatures associated with GMV changes in adolescents with ADHD.

**Methods:**

Voxel-based morphometry was performed on structural MRI data from 27 adolescents with ADHD and 34 typically developing (TD) controls to map regional GMV differences. The spatial pattern of these alterations was then correlated with whole-brain gene expression profiles from the Allen Human Brain Atlas using partial least squares (PLS) regression. Functional and cell-type enrichment analyses were conducted on significant gene sets. Finally, three machine-learning models (support vector machine, random forest, and decision tree) were developed to evaluate the diagnostic utility of GMV changes.

**Results:**

Increased GMV was observed in the bilateral precuneus, and decreased GMV was found in the left middle occipital gyrus and orbital part of the right inferior frontal gyrus in ADHD compared to TD. The spatial distribution of these GMV changes was significantly correlated with a specific gene expression pattern. Functional enrichment analysis revealed that positively correlated genes were involved in fundamental cellular processes, while negatively correlated genes were associated with synaptic organization and brain development. Cell-type analysis demonstrated significant enrichment of positively correlated genes in microglia, and negatively correlated genes in excitatory and inhibitory neurons. Random Forest achieved the highest accuracy in distinguishing between ADHD and TD (AUC = 0.871 ± 0.029).

**Conclusion:**

In summary, this study provides unique insights into the brain structural development of attention-deficit/hyperactivity disorder (ADHD) and offers new perspectives for the future diagnosis and treatment of ADHD.

## 1 Introduction

Attention-Deficit/Hyperactivity Disorder (ADHD) is a prevalent neurodevelopmental disorder characterized by a persistent pattern of inattention, hyperactivity, and impulsivity that is inconsistent with developmental level. With an estimated global prevalence of approximately 3.4% in children, ADHD profoundly impairs academic performance, social functioning, and family life (1). Its impact frequently persists into adulthood, leading to elevated risks of psychiatric comorbidities, occupational difficulties, and poor social adaptation, thereby constituting a significant public health burden. (2). Although its etiology is complex, involving interactions between genetic and environmental factors, ADHD is generally understood to originate from atypical brain development in both structure and function. (3).

In recent years, neuroimaging techniques, particularly structural magnetic resonance imaging, have provided direct evidence for elucidating the neurobiological underpinnings of ADHD. Numerous voxel-based morphometry studies consistently report significant reductions in gray matter volume (GMV) across multiple regions in children with ADHD compared to typically developing peers. These abnormalities are predominantly localized to the prefrontal cortex, basal ganglia (including the caudate nucleus and putamen), cerebellum, and certain temporal lobe regions—areas collectively forming critical neural networks that subserve attentional control, executive functions, motivation regulation, and motor inhibition. However, heterogeneity remains in the existing literature; for instance, the direction of change in certain structures such as the amygdala and hippocampus continues to be debated, and specific associations between regional gray matter alterations and distinct clinical dimensions (inattention vs. hyperactivity/impulsivity) have yet to be fully elucidated. (4–6). Importantly, conventional neuroimaging research remains largely correlative and cannot explain the microscopic molecular mechanisms underlying these macroscopic structural abnormalities, thereby limiting deeper insight into the pathophysiology of ADHD (7).

Rapid advances in transcriptomic technologies now offer a transformative tool for bridging macro-scale brain structure and micro-scale molecular mechanisms. By analyzing postmortem human brain samples or leveraging comprehensive gene expression databases such as the Allen Human Brain Atlas, researchers can map the spatial expression patterns of genes across the brain (8, 9). This has given rise to an emerging analytical approach: cross-modal spatial correlation analyses that integrate neuroimaging-derived maps of gray matter abnormalities with whole-brain gene expression profiles. Such imaging–transcriptomics association studies systematically investigate whether the structural alterations observed in ADHD are linked to specific genes, cell types, or biological pathways. Recent work in depression and schizophrenia has demonstrated that cortical structure correlates with distinct gene sets and cell types (10, 11). Nevertheless, parallel research in pediatric ADHD populations remains scarce.

Therefore, the present study was designed to integrate neuroimaging and transcriptomic data to advance our understanding of the relationship between molecular mechanisms and structural changes in ADHD. First, voxel-based morphometry was employed to identify disorder-specific patterns of GMV alterations. This was followed by the use of partial least squares regression to examine whether these gray matter changes spatially correlate with specific gene expression patterns. Third, enrichment analyses were performed on the identified gene sets to delineate associated biological pathways and cell types. Finally, machine learning (ML) was used to explore the potential of these GM volume alterations in differentiating between ADHD and TD.

## 2 Methods

### 2.1 Study participants

This study utilized data from the Penn Longitudinal Executive functioning in Adolescent Development (Penn LEAD) study, comprising 27 individuals diagnosed with ADHD and 34 typically developing (TD) controls without a current or lifetime history of any DSM-5 diagnosis. The protocol of this study records the inclusion and exclusion criteria and flowchart in detail (12).

### 2.2 MRI acquisition

A comprehensive description of the parameters can be found in Sevchik BL et al. (12). In short, MRI data were acquired on a 3T Siemens Prisma scanner (Erlangen, Germany) with a 32-channel head coil at the University of Pennsylvania. Participants' T1-weighted structural images were acquired using parameters closely matching those employed in the Adolescent Brain Cognitive Development (ABCD) study (176 slices; TR = 2,500 ms; TI = 1,070 ms; echo time, TE = 2.9 ms; flip angle, FA = 8°; field of view, FOV = 256 x 256 mm; matrix size = 256 x 256; voxel size = 1.0 mm; phase encoding direction = A >> P; acquisition time 7:12 min).

### 2.3 MRI preprocessing

For all pre-processing steps, the default parameters as implemented in CAT12 (Computation Anatomy Toolbox for SPM, build 1184, Christian Gaser, Structural Brain Mapping Group, Jena University Hospital, Germany, http://dbm.neuro.uni-jena.de/cat/) were employed. Detailed preprocessing steps can be found in the Supplementary materials. After all preprocessing steps are completed, the segmented gray matter images were smoothed using a 6 × 6 × 6 mm^3^ full-width-at-half maximal Gaussian kernel. For statistical testing, we used a general linear model (GLM) to examine the differences between the ADHD and TD groups, with group as the factor of interest and age, gender, and total intracranial volume (TIV) included as covariates. Multiple comparisons were corrected using a voxel-wise false discovery rate (FDR) method.

### 2.4 Gene expression data preprocessing

The available gene expression data of six postmortem brains with 3,702 distinct samples were provided by the AHBA database (13). We employed the Schaefer 300 parcellation atlas to map the transcriptomic data onto the cerebral cortex (14). The AHBA dataset was processed according to Arnatkevic et al. (15). In summary, genetic probes were reannotated rather than relying on the default probe information in the Allen Human Brain Atlas (AHBA) dataset, thereby discarding probes that could not be reliably matched to genes. In accordance with previously published guidelines for probe-to-gene mapping and intensity-based filtering, probes with expression levels below background noise in at least 50% of samples were excluded. Tissue samples were assigned to brain regions based on their adjusted MNI coordinates (https://github.com/chrisfilo/alleninf), with each sample matched to the nearest region within a 2 mm radius. To minimize misassignment, matching was constrained by hemisphere and by cortical vs. subcortical division. If a brain region could not be assigned to any sample through the above procedure, the sample closest to the centroid of that region was selected, ensuring that all regions were assigned a value. Samples assigned to the same brain region were averaged per donor. Gene expression values for each donor were normalized using a robust sigmoidal function and rescaled to the unit interval. Expression profiles were then averaged across all donors to generate a single matrix, with rows representing brain regions and columns corresponding to the 15,745 retained genes. Because the AHBA dataset included only two right hemisphere data, only the left hemisphere was considered in our analysis. Thus, a mean of all samples in a region was calculated to obtain the matrix (150 regions × 15,745 gene expression levels) of transcriptional level values.

### 2.5 Identifying transcriptional correlates of cortical GM volume alterations in ADHD

Partial least squares (PLS) (16) regression was employed to characterize the association between regional alterations in gray matter (represented by *t*-values derived from 150 cortical regions in the left hemisphere) and transcriptional activity across all 15,745 genes. In this model, gene expression data served as the predictor variables for the regional changes in gray matter volume. The first PLS component (PLS1) represents the linear combination of gene expression values that exhibited the strongest correlation with the pattern of regional gray matter volume change. To assess the statistical significance of PLS1, permutation testing (*n* = 5,000 iterations) incorporating spherical rotations of the gray matter volume map was performed to account for spatial autocorrelation. This tested the null hypothesis that PLS1 explained no more covariance between the gray matter volume map and the whole-genome expression profile than would be expected by chance. Bootstrapping was subsequently applied to estimate the variability of each gene's weight within PLS1. The ratio of each gene's weight to its bootstrap-estimated standard error was used to derive Z scores, which ranked genes according to their contribution to PLS1. Genes with a false discovery rate (FDR) < 0.05—constituting either a positive (PLS1+) or negative (PLS1–) association—were defined as the final gene set associated with regional gray matter volume changes.

### 2.6 Enrichment analysis

We performed functional enrichment analysis using Metascape (https://metascape.org/). Gene lists were analyzed for significant enrichment in Gene Ontology (GO) biological processes and Kyoto Encyclopedia of Genes and Genomes (KEGG) pathways (17). A significance threshold of 5%, corrected for the false discovery rate (FDR), was applied to all enrichment analyses.

### 2.7 Assigning ADHD-related genes to cell types

To obtain gene sets from each cell type, we compiled data from five different single-cell studies using postmortem cortical samples of human postnatal participants (18). This approach avoids bias based on acquisition methodology, analysis, or thresholding, and led to the initial inclusion of 58 cell classes, many of which were overlapping based on nomenclature and/or constituent genes. Moreover, these cell type-specific gene sets demonstrate biological consistency on the spatial maps of AHBA. Referring to previous studies, we organized cell types into seven canonical classes: microglia, endothelial cells, oligodendrocyte precursors, oligodendrocytes, astrocytes, excitatory, and inhibitory neurons. Two studies did not subdivide neurons into excitatory and inhibitory sets, and thus these gene sets were excluded from this cell-class assignment. To assign ADHD-related genes obtained by PLS analysis to cell types, we overlapped the gene set of each cell type with the PLS1– rank gene list. The *p*-value of the number of overlapped genes in each cell type was obtained by a permutation test, and corrected by FDR with *p* < 0.05.

Furthermore, to ensure the robustness of the results, we repeated the cell type-specific analysis using only the three studies that provided excitatory/inhibitory neuron classifications.

### 2.8 Machine learning validation of classification performance

To further explore whether GMV can serve as an effective biomarker, we extracted the classification features from brain regions that showed significant changes in GMV in the ADHD group compared to the TD group. These were combined with three demographic covariates (age, sex, and total intracranial volume (TIV)) to form a 7-dimensional feature vector for each participant (*n* = 61: 27 ADHD, 34 TD controls). Three classifiers were implemented: support vector machine (SVM) with radial basis function kernel, Random Forest, and Decision Tree. To prevent data leakage, feature selection (ANOVA *F*-test, *k* = 5) and z-score normalization were performed independently within each cross-validation fold. Models were trained with class-weighted learning to address class imbalance. Hyperparameters were fixed (SVM: *C* = 1.0, gamma = ‘scale'; random forest: 100 estimators, max_depth = 5; gradient boosting: 100 estimators, max_depth = 3, learning_rate = 0.1) based on pilot analyses. Performance was assessed through 50 repetitions of 5-fold stratified cross-validation, with different random partitions in each repetition. We reported the area under the receiver operating characteristic curve (AUC), sensitivity, specificity, and balanced accuracy. Metrics are presented as mean ± standard deviation across all repetitions, with 95% confidence intervals calculated via percentile bootstrap.

## 3 Results

### 3.1 Demographic and clinical data

The ADHD and TD groups were comparable in terms of age, sex, gray matter volume, and TIV. The clinical and demographic information is summarized in Table 1.

**Table 1:** Demographic and clinical variables.

Variables	ASHD (*N* = 27)	TD (*N* = 34)	*P*-values
Age, mean (SD)	11.0 (2.0)	11.2 (2.1)	0.615
Female, *n* (%)	14 (51.9%)	13 (38.2%)	0.288
Gray matter volume (SD)	766.4 (88.1)	792.74 (73.1)	0.235
Total intracranial volume (SD)	1397.5 (151.8)	1442.1 (137.5)	0.207

### 3.2 Differences in gray matter volume between ADHD and TD groups

The GLM analysis revealed significant alterations in GMV in individuals with ADHD. Compared to the typically developing (TD) group, individuals with ADHD exhibited significantly increased GMV in the bilateral precuneus, whereas significant volume reductions were observed in the left middle occipital gyrus and the orbital part of the right inferior frontal gyrus. Detailed information regarding these regions of significant difference is presented in Table 2 and Figure 2.

**Table 2:** Gray matter volume alterations in brain regions between the ADHD and TD groups.

Brain regions	MNI coordinates			Peak *T*-value	Cluster size
	X	Y	Z		(voxels)
ADHD > TD					
Right precuneus	24	−50	18	5.39	291
Left precuneus	−24	−62	24	5.08	186
ADHD < TD					
Left middle occipital gyrus	−24	−60	36	5.28	368
Right orbital inferior frontal gyrus	34	36	−8	4.64	190

ADHD, attention-deficit/hyperactivity disorder; TD, typically developing.

**Figure 1:**
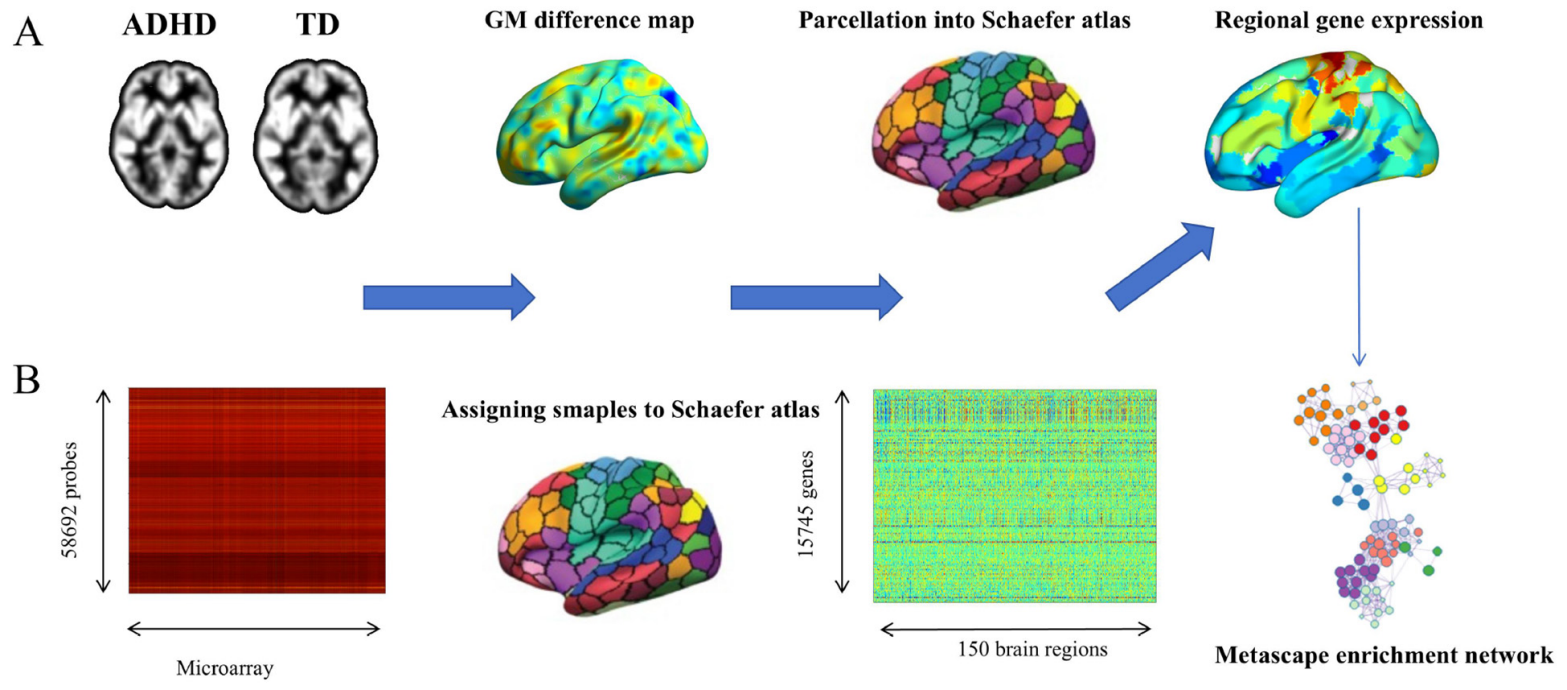
Study overview. **(A)** Gray matter volume analysis. Two-sample *t*-tests were used to obtain threshold-free *T*-value maps of gray matter differences in ADHD, which were then projected onto the Schaefer atlas. **(B)** Gene expression analysis. Gene expression data from 152 regions of the left hemisphere were obtained from the Allen Human Brain Atlas and averaged across six postmortem donors. Partial least squares (PLS) regression was applied to identify associations between transcriptomic patterns and neuroimaging measures. Finally, enrichment analysis was conducted on the set of genes most strongly associated with the first PLS component.

**Figure 2:**
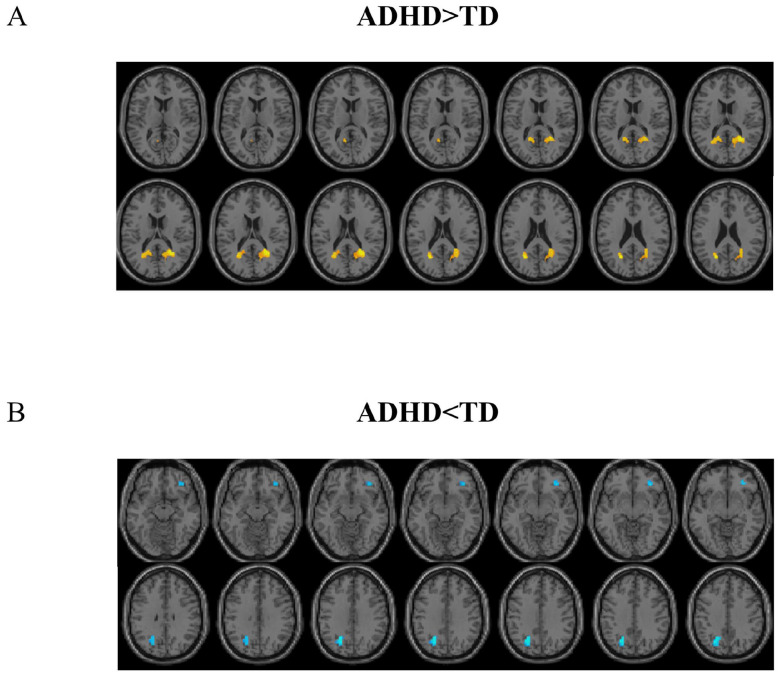
GMV analysis results. **(A)** Regions of increased gray matter volume in ADHD. **(B)** Regions of reduced gray matter volume in ADHD.

### 3.3 Gene expression associated with ADHD-related gray matter volume changes

**Figure 3:**
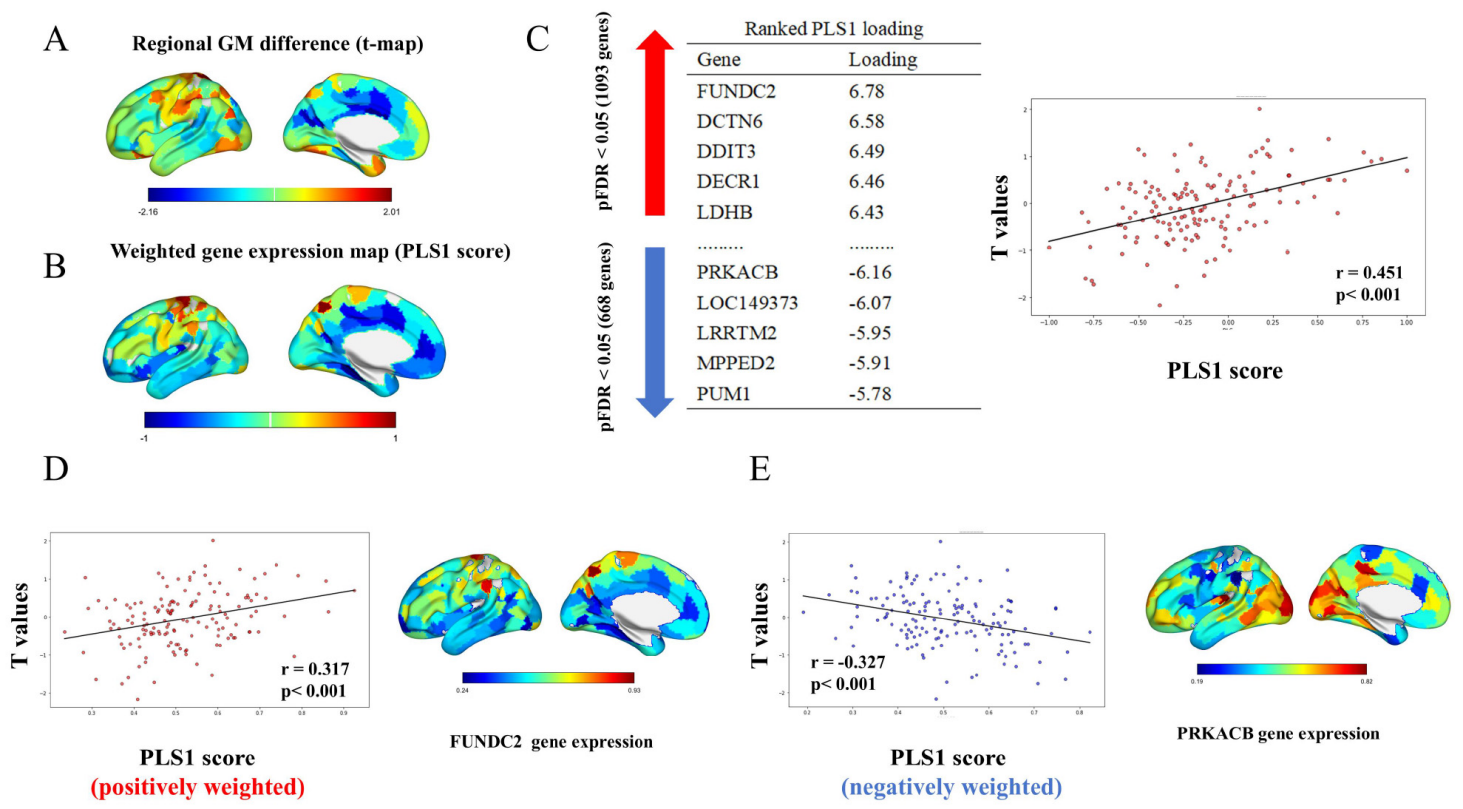
Gene expression profiles related to gray matter volume differences. **(A)** ADHD gray matter volume difference *T*-value map (unthresholded). **(B)** A weighted gene expression map of regional PLS1 scores in the left hemisphere (unthresholded). **(C)** Ranked PLS1 loadings and a scatterplot of regional PLS1 scores (a weighted sum of 15,745 gene expression scores) and regional changes in gray matter volume (*r* = 0.451, *p* < 0.001). **(D)** Genes that are strongly positively weighted on PLS1 (e.g., FUNDC2) correlate positively with ADHD gray matter volume difference *T*-value map (*r* = 0.317, *p* < 0.001). **(E)** Genes that are strongly negatively weighted on PLS1 (e.g., PRKACB) correlate negatively with ADHD gray matter volume difference *T*-value map (*r* = −0.327, *p* < 0.001).

We employed PLS analysis to identify gene expression patterns associated with alterations in gray matter volume in ADHD. PLS1 accounted for the largest proportion of variance in gray matter volume (37.7%) and demonstrated statistical significance at a probabilistic threshold (*p*-boot = 0.001). PLS1 scores showed a positive correlation with the intergroup t-map (*r* = 0.451, *p*-spin < 0.001). Our analysis revealed 1,093 PLS1+ genes (*Z* > 2.3, pFDR < 0.05), whose expression levels are positively correlated with increases in gray matter volume, and 668 PLS1– genes (*Z* < −2.3, pFDR < 0.05), whose expression levels are negatively correlated with such increases (see in Figure 3)

### 3.4 Gene set enrichment

**Figure 4:**
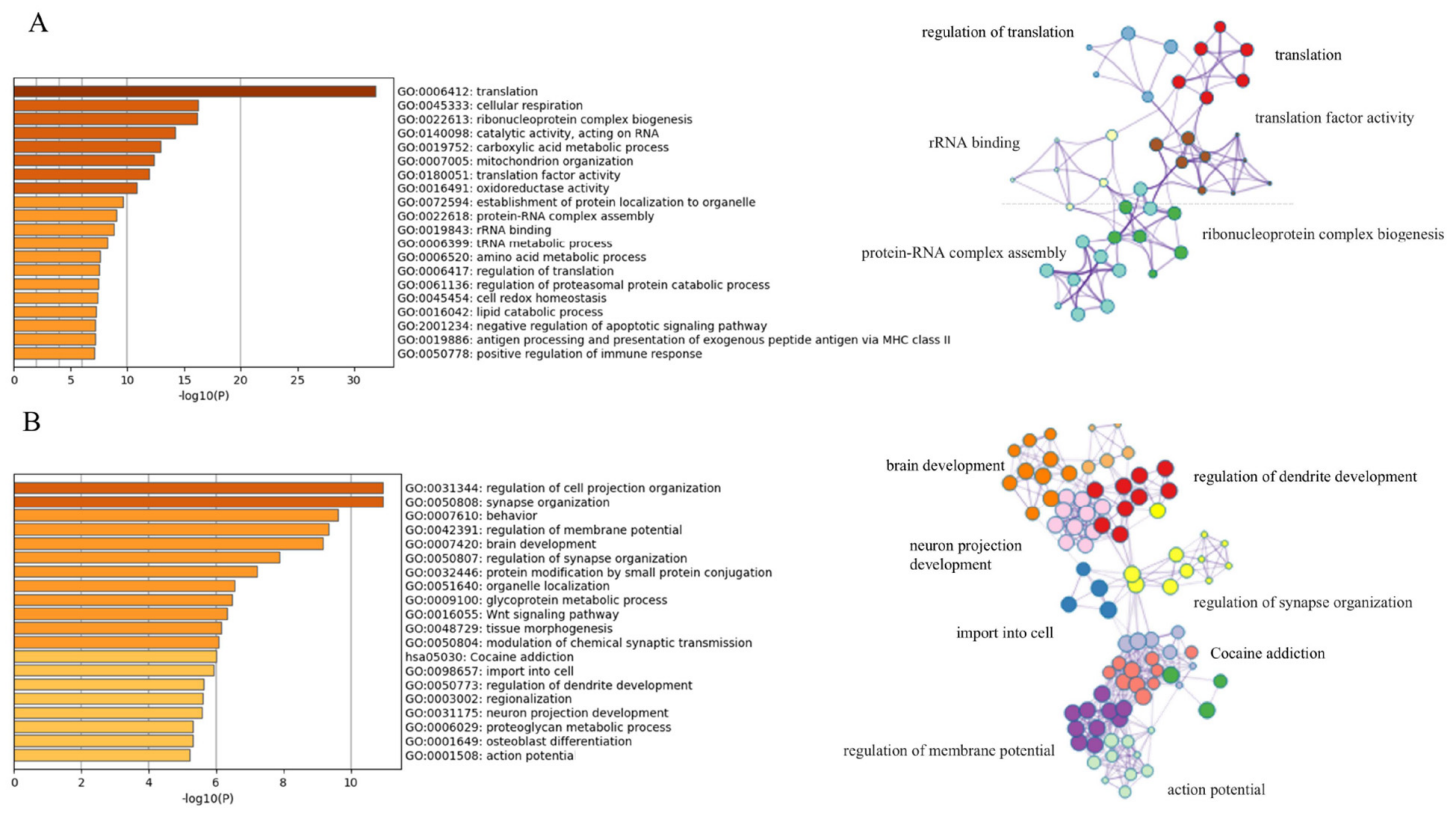
Functional enrichment of gene transcripts. **(A)** Results of PLS+ functional enrichment analysis. **(B)** Results of PLS- functional enrichment analysis. More detailed results of the Metascape enrichment network analysis are shown in Fig. S1.

We identified the top 20 enriched pathways. Significantly enriched GO biological processes for PLS+ were predominantly associated with “translation,” “cellular respiration,” “ribonucleoprotein complex biogenesis,” “catalytic activity, acting on RNA,” and “mitochondrion organization.” In contrast, PLS– genes were primarily linked to “regulation of cell projection organization,” “synapse organization,” “behavior,” “regulation of membrane potential,” and “brain development.” The functional enrichment results are presented in Figure 4 and Supplementary Figure S1.

### 3.5 Cell types are associated with alterations in cortical gray matter volume

**Figure 5:**
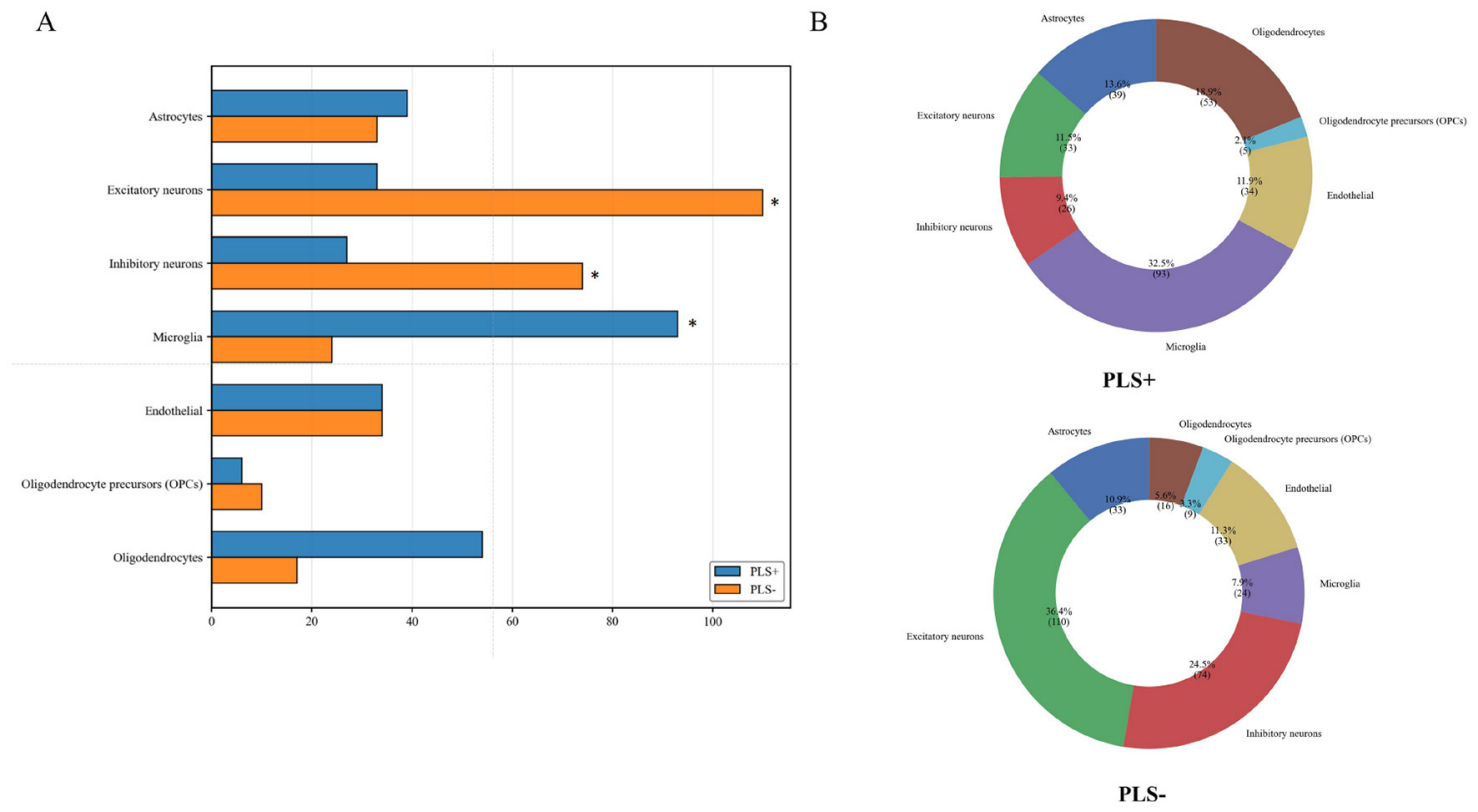
Cell type-specific expression to changes in GMV-related genes. **(A)** The number of overlapping genes for each cell type. **(B)** The proportion of overlapping PLS+ and PLS- genes distributed across all cell types.

Cell type analysis revealed that PLS+ genes were predominantly enriched in microglia (number = 93, *P*_FDR_ < 0.05), whereas PLS– genes showed primary enrichment in excitatory (number = 110, *P*_FDR_ < 0.05) and inhibitory neurons (number = 74, *P*_FDR_ < 0.05). These results are presented in Figure 5. The results of the sensitivity analysis are consistent with the main analysis: apart from excitatory and inhibitory neurons, only PLS+ genes are primarily enriched in microglia (count = 26, PFDR < 0.05) (Supplementary Figure S2).

### 3.6 The performance of machine learning–based model

**Figure 6:**
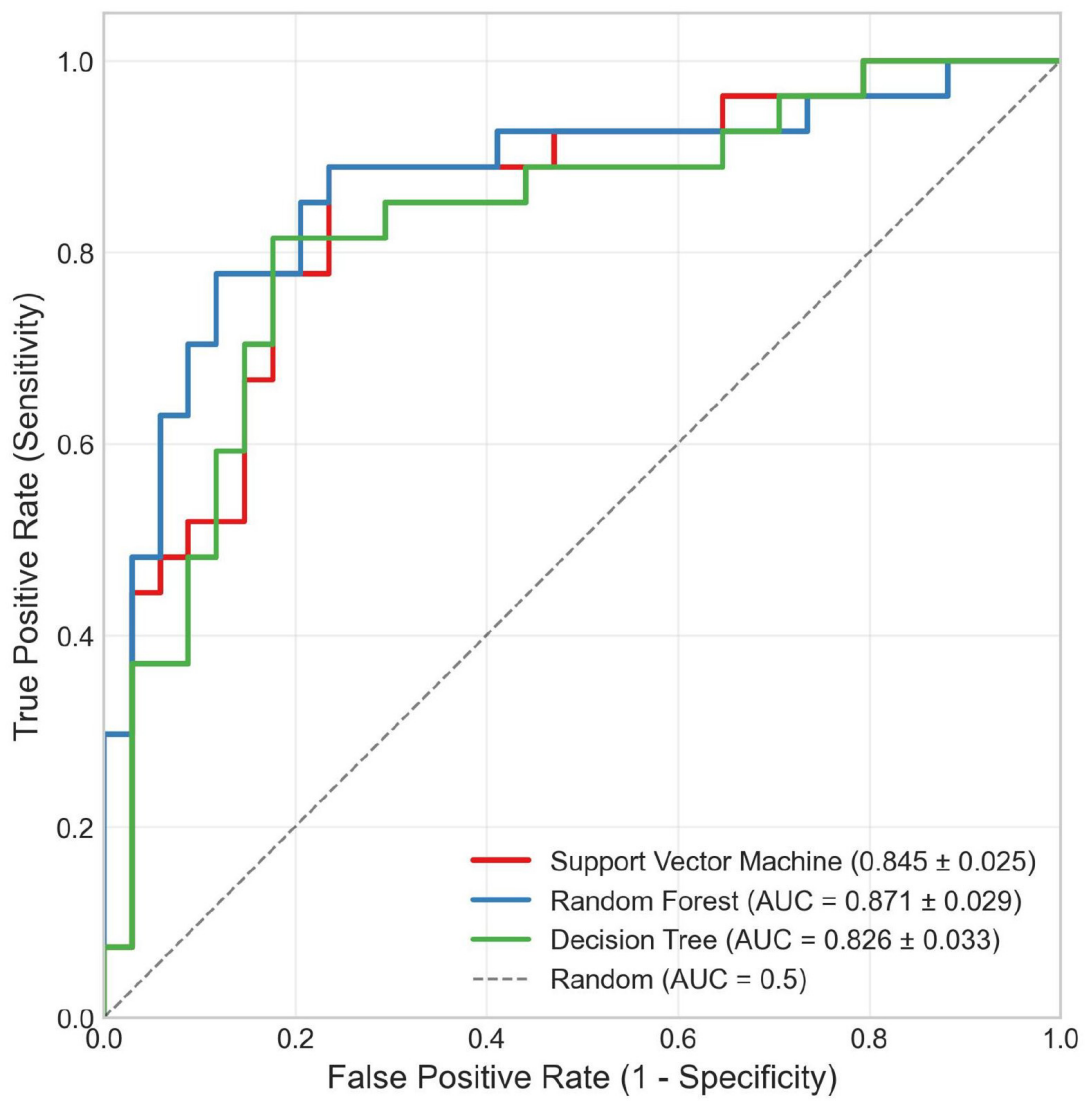
The receiver operating characteristic (ROC) curves of three machine learning models. AUC, area under curve.

The performance summaries of the three machine learning models are shown in Figure 6 and Table SX. Among them, the Random Forest model demonstrated the strongest classification performance (AUC = 0.871 ± 0.029), followed by SVM (AUC = 0.845 ± 0.025) and Decision Tree (AUC = 0.826 ± 0.033), which exhibited relatively lower classification performance. (See in Figure 6 and Supplementary Table S1)

## 4 Discussion

This study integrates structural neuroimaging with transcriptomic profiling to elucidate the molecular and cellular correlates underlying GMV alterations in adolescents with ADHD. We identified a spatially patterned profile of GMV changes, characterized by increases in the bilateral precuneus and reductions in the left middle occipital gyrus and the orbital part of the right inferior frontal gyrus. Crucially, the spatial distribution of these macroscale alterations was significantly associated with the gene expression patterns in specific brain regions. This transcriptional signature was not random but pointed to the enrichment of distinct biological processes and, most importantly, specific cell types (19). Our findings provide a novel, multi-scale framework for understanding ADHD pathophysiology, linking regional brain structural deficits to their underlying molecular and cellular mechanisms.

The primary finding of this study is the cell type-specific enrichment of the ADHD-related GMV transcriptional signature. Genes whose expression patterns positively correlated with the GMV alteration map (PLS1+) were predominantly expressed in microglia (20, 21). The positive correlation between microglial gene expression and increased GMV contrasts with the more common association of neuroimmune activation with tissue loss. This suggests that, within the context of adolescent development, the microglial transcriptomic signature we identified may relate less to a degenerative state and more to their essential roles in synaptic refinement and circuit sculpting. Specifically, in ADHD, this signature could reflect an alteration in the timing, precision, or extent of activity-dependent synaptic pruning. This may lead to a relative increase or a delay in the normative reduction of GMV in regions such as the precuneus, implying that the observed GMV increase signifies a lack of optimal synaptic refinement rather than a protective or proliferative process. This interpretation aligns with our complementary finding that GMV reductions were linked to neuronal synaptic gene expression (PLS1-). Together, these findings point toward a convergent pathology centered on disrupted synaptic homeostasis and circuit maturation, strongly implicating neuroimmune mechanisms and specifically positioning microglial function as a key player in shaping the atypical neurodevelopment characteristic of ADHD (22).

Conversely, genes whose expression was negatively correlated with the GMV alteration map (PLS1–) were primarily enriched in excitatory and inhibitory neurons. These genes were associated with synaptic organization, regulation of membrane potential, and brain development (23). This negative correlation suggests that in brain regions where GMV is reduced in individuals with ADHD, gene programs related to synaptic function and neuronal communication are actually relatively highly expressed. This may reflect compensatory activation, abnormal neural plasticity, or homeostatic dysregulation during development in these regions, rather than simply impaired function. Alternatively, the higher expression of these associated genes might indicate that the region is in an immature or inefficient state of synaptic pruning and reorganization, ultimately leading to the observed macroscopic reduction in GMV. This finding—specifically, the abnormally elevated expression of synaptic function-related genes in brain regions with reduced GMV—provides new molecular-level support for, yet also indicates a more complex mechanism underlying, the long-standing hypothesis that ADHD involves deficits in synaptic signaling and neural circuit efficiency. The co-enrichment of these genes in both excitatory and inhibitory neurons points to a widespread disruption in the delicate balance of cortical excitation and inhibition, which is essential for normal attention, cognitive control, and motor functioning—core domains impaired in ADHD. The observed upregulation of genes associated with synaptic organization may not reflect enhanced function, but rather dysregulation of synaptic homeostasis, disruption of neural circuit maturation, or inefficient compensatory remodeling—aberrant processes that may ultimately impair the overall efficiency of neural signaling (24, 25).

The spatial pattern of GMV alterations itself is noteworthy. The observed reduction in the orbitofrontal cortex is consistent with extensive literature implicating prefrontal dysfunction in ADHD's executive and inhibitory deficits (26). The increase in GMV within the precuneus, a key node of the default mode network (DMN), may reflect a failure of typical maturational trajectories, such as synaptic pruning (27). This could underlie the difficulty in suppressing DMN activity during goal-directed tasks, a proposed mechanism for attentional lapses in ADHD. The involvement of the occipital gyrus suggests that visual processing circuits may also be part of the broader neurodevelopmental atypicality in ADHD, a area deserving of further investigation (28).

The high classification accuracy (AUC = 0.891) achieved by our SVM model demonstrates the potential utility of these GMV alterations as a neuroanatomical biomarker. It confirms that the identified structural differences, though spatially specific, are robust enough to distinguish individuals with ADHD at a group level (29). This moves beyond mere correlation and suggests a degree of diagnostic relevance, paving the way for future research to explore the prognostic or treatment-response predictive value of such imaging markers (30).

Our study has several limitations. First, the sample size, while adequate for the primary analyses, is modest, and replication in larger, independent cohorts is essential. Second, the transcriptomic data from the Allen Human Brain Atlas, while an invaluable resource, derive from a small number of adult postmortem brains. This limits direct inferences about developmental changes in gene expression during adolescence, a critical period for ADHD. Future studies incorporating age-specific transcriptomic data will be important. Third, the cross-sectional design of this study warrants future validation in longitudinal cohorts.

In conclusion, our integration of macrostructural neuroimaging and microstructural transcriptomics demonstrates that gray matter volume alterations in ADHD are associated with cell-type-specific transcriptomic signatures. These signatures prominently implicate microglial and neuronal cell types, suggesting a convergent pathology involving neuroimmune mechanisms and synaptic dysfunction. Together, our findings provide unique insights into the mechanisms underlying aberrant gray matter development in ADHD and open new avenues for therapeutic intervention.

## 5 Conclusion

In summary, we identified an abnormal GMV development model in ADHD, which can serve as a potential biomarker for the classification and prediction of the disorder. We further linked the GMV abnormality patterns to gene expression levels. This study provides unique insights into brain structural development in ADHD and offers new perspectives for future ADHD treatment.

## Data Availability

Publicly available datasets were analyzed in this study. This data can be found here: The data from the Penn Longitudinal Executive Functioning in Adolescent Development (Penn LEAD) study used in this research are publicly available on OpenNeuro (https://openneuro.org/datasets/ds007116). All supporting data (e.g., statistical/analytic code) are available upon reasonable request.
