# Supplementary material for: Gray matter volume alterations in adolescents with ADHD are associated with cell type-specific transcriptional signatures
Source: Front Neurol. 2026 May 29;17:1815799. doi: 10.3389/fneur.2026.1815799 (PMC13260793; doi:10.3389/fneur.2026.1815799)
Supplement: Supplementary file 1 [file Supplementary_file_1.docx]

**MRI preprocess**

In an initial voxel-based processing step, a spatial adaptive non-local means (SANLM) denoising filter was applied, followed by internal resampling to integrate low-resolution images and anisotropic spatial resolutions. After the correction of intensity inhomogeneity in T1-weighted images, resulting from inhomogeneities of the magnetic field, also referred to as bias correction, T1 weighted images were segmented into white matter (WM), grey matter (GM) and cerebrospinal fluid (CSF), using the standard unified segmentation. In a refined voxel-based processing step, skull-stripping and regional parcellation of the brain into left and right hemisphere, cerebellum and subcortical areas were performed on the segmented data and possible local white matter hyperintensities were detected. Subsequently, a local intensity transformation of all tissue classes was conducted, followed by a final maximum a posteriori estimation of tissue segmentation. In order to estimate the amount of each tissue type per voxel, a partial volume estimation was employed. Finally, images were spatially normalized using DARTEL. This involved registering all images to the same template by estimating a 12-parameter affine transformation.


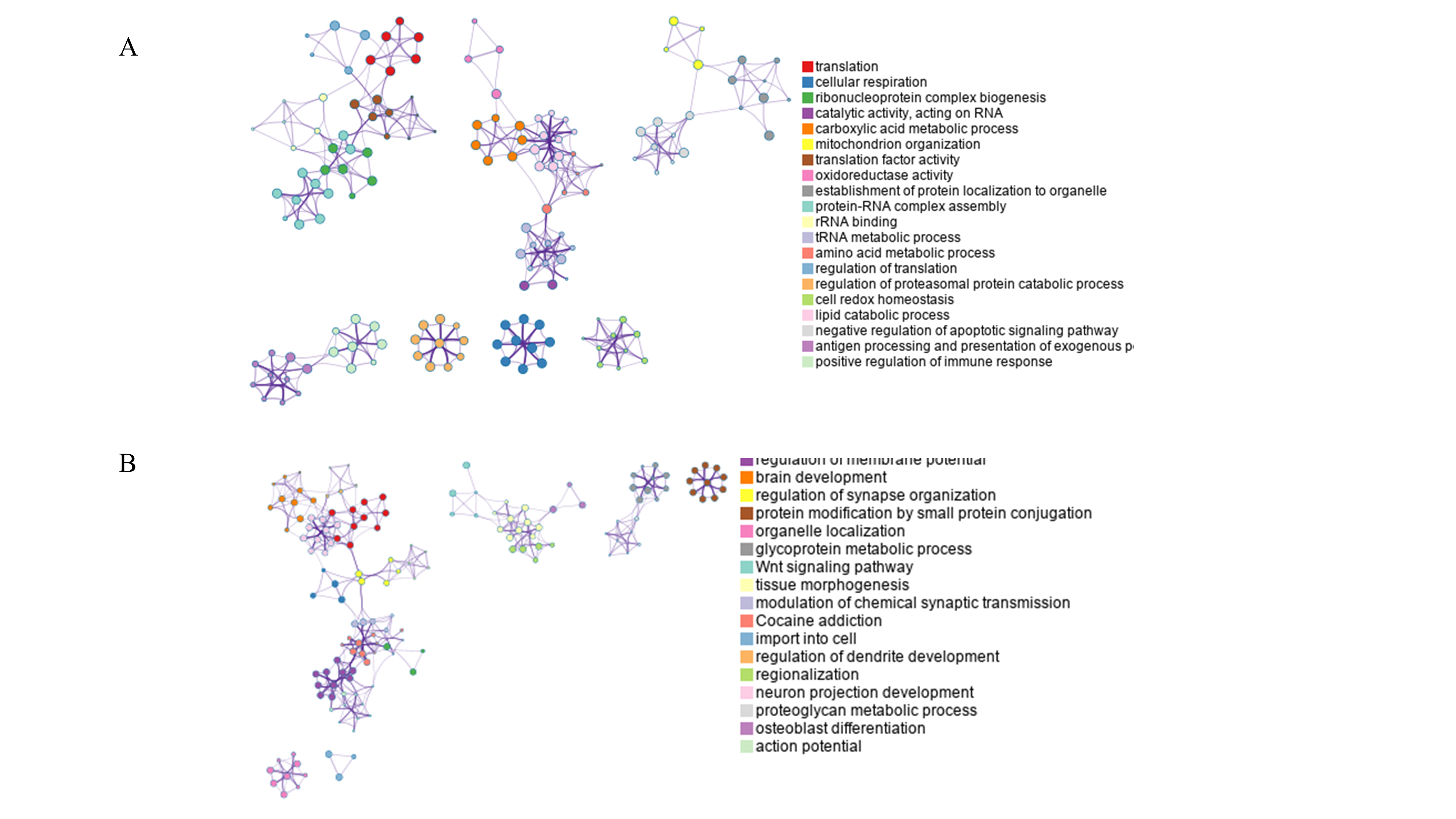


Fig. S1 Metascape enrichment network visualization. A: PLS+ Metascape enrichment network result. B: PLS- Metascape enrichment network result.

| Table S1: Model performance metric results for three machine learning models. | | | |
| --- | --- | --- | --- |
| Model performance metrics. | SVM | Random Forset | Decision Tree |
| AUC | 0.845 ± 0.025 | 0.871 ± 0.029 | 0.826 ± 0.033 |
| Sensitvity | 0.842 ± 0.046 | 0.788 ± 0.045 | 0.731 ± 0.064 |
| Specificity | 0.791 ± 0.046 | 0.833 ± 0.034 | 0.821 ± 0.047 |
| Balanced Acc | 0.816 ± 0.034 | 0.811 ± 0.030 | 0.776 ± 0.041 |


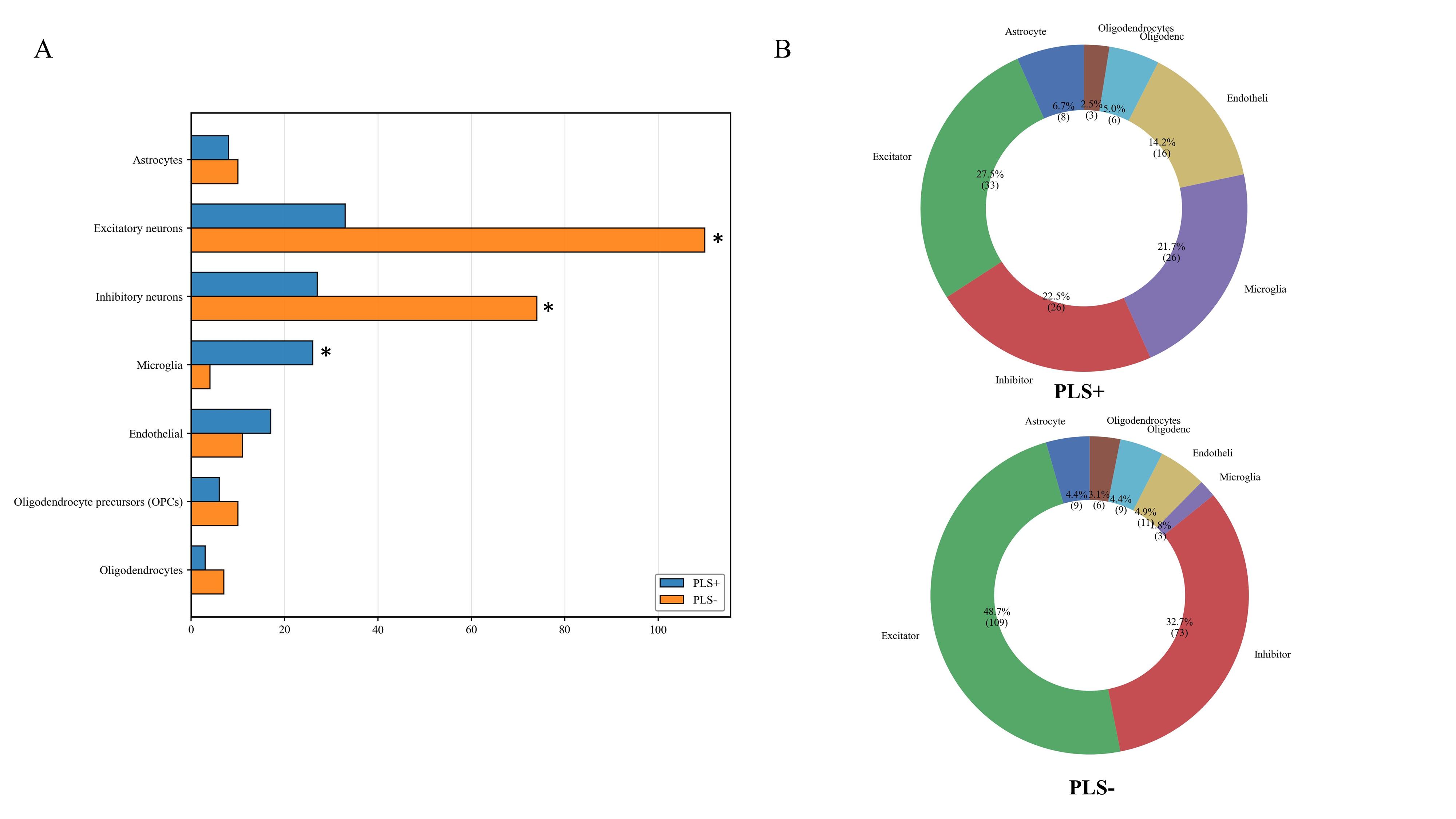
Fig. S2 The results of the cell type-specific analysis using only the three studies that provided excitatory/inhibitory neuron classifications.
